# The Effect of Hemp Cake (*Cannabis sativa* L.) on the Characteristics of Meatballs Stored in Refrigerated Conditions

**DOI:** 10.3390/molecules26175284

**Published:** 2021-08-31

**Authors:** Klaudia Kotecka-Majchrzak, Natalia Kasałka-Czarna, Anita Spychaj, Beata Mikołajczak, Magdalena Montowska

**Affiliations:** Department of Meat Technology, Poznan University of Life Sciences, Wojska Polskiego 31, 60-624 Poznan, Poland; klaudia.kotecka@up.poznan.pl (K.K.-M.); natalia.czarna@up.poznan.pl (N.K.-C.); anita.spychaj@up.poznan.pl (A.S.); beata.mikolajczak@up.poznan.pl (B.M.)

**Keywords:** meat, hemp cake, physicochemical quality, oxidation, sensory evaluation

## Abstract

Hemp cake, a by-product of cold pressing oil from hemp seeds, is a nutritious ingredient that could be used for the production of new or reformulated meat products. The aim of this study was to determine the influence of inclusion of 0.9%, 2.6%, 4.2%, and 7.4% (*w*/*w*) hemp cake (*Cannabis sativa* L.) on the physicochemical and textural properties, oxidation, and sensory acceptance of cooked and vacuum-packed meatballs during refrigerated storage. The addition of 7.4% hemp cake enhanced the amount of dry matter and reduced the content of water. Lightness (L*) and redness (a*) values reduced significantly with higher levels of hemp supplementation. Regardless of the amount of hemp additive, pH, color parameters did not differ significantly during the 12 days of storage. Hemp cake significantly decreased protein and lipid oxidation: the inhibitory effect of adding 7.4% hemp cake on protein carbonyl group formation and TBARS values reached 11.16% and 36.5%, respectively, after 10 days of storage. Sensory analysis revealed that meatballs prepared with 0.9% and 2.6% hemp cake gained higher overall scores. The results indicate that hemp cake, a material considered mainly as waste, may be destined for food purposes and be an alternative ingredient for the production of sustainable meat products.

## 1. Introduction

World meat consumption continues to increase, driven by a growing population and greater demand for meat in Europe, North America, and Asian and Latin American countries. Statistics predict that this trend will continue. The highest meat consumption per capita from 2016 to 2018 was recorded in North America (95.3 kg), it was 64.8 kg in Europe, and will increase in 2028 to 97.1 kg and 66.8 kg, respectively. The growing consumption applies mainly to poultry meat and pork and, to a lesser extent, beef and veal, and sheep and goat meat [1]. Simultaneously, in developed countries, the meat product market is becoming more and more diversified, since some consumers are increasingly seeking enriched and functional meat products that would positively influence their health. Therefore, technologists and producers are more willing to modify or reformulate meat products, for example by replacing part of the fat-meat fraction with a vegetable fraction. For this purpose, various types of oleaginous seeds are used, in the form of whole seeds, flours, refined and unrefined oils, and oil-pressing by-products including oilseed cake, meal, and protein isolates or hydrolysates derived from, for instance, black cumin seeds, flax, pumpkin, sesame, and sunflower seeds [2,3,4,5]. In recent years, there has also been growing interest in the use of hemp seeds and hemp oil in feed production and vegan foods, as well as the enrichment of bakery and dairy food products with hemp ingredients, but only few research has been dedicated to meat products [6,7,8,9].

Hemp (*Cannabis sativa* L.) is an annual plant indigenous to Asia but now low-drug industrial varieties of hemp can be legally cultivated worldwide. According to the European Union agricultural policy, hemp production can be eligible for support if the varieties used have a THC (delta-9-tetrahydrocannabinol—the main psychoactive substance of cannabis) content not exceeding 0.3% (Regulation EU No 1307/2013). On 23 October 2020, the European Parliament raised the authorized THC level for industrial hemp from 0.2% to 0.3%, bringing it in line with American policy (P9_TA-PROV(2020)0287, Amendment 8). Whereas the European Union court of justice made a decision not to treat cannabidiol (CBD) as a narcotic drug. The growing interest in hemp seeds results from their nutritional and pro-health properties [10]. Hemp seeds are a good source of protein (25–30%), polyunsaturated fatty acids (PUFA; 75–80%), fiber (~4%), and the percentage distribution of essential amino acids is similar to that in soybean but hemp seeds contain more methionine [8,11]. Hemp seed proteins hydrolyzed with both gastrointestinal enzymes, i.e., pepsin, pancreatin, trypsin, and commercial proteases, release bioactive peptides with antihypertensive, antioxidant, anti-proliferative, and anti-inflammatory activities [12,13,14,15].

The use of hemp seed flour as a substitute for milk, wheat, and meat protein has been evaluated in bakery and extruded products, infant formula, and yogurt [8,16,17]. There are also vegan and vegetarian products available on the market with a declared content of up to 10% hemp seeds. In relation to meat products, only a few studies have been published, i.e., chopped semi-finished beef cutlets with 5–15% of the minced beef replaced with hemp flour, as well as pork loaves with the addition of 5% hemp seeds, de-hulled hemp seeds, hemp flour, and hemp protein showed an improvement in some textural, nutritional, and sensory properties [9,18]. Recently, the replacement of soy protein isolate (SPI) with hemp protein concentrate in high-moisture meat analogs produced by extrusion was investigated [19]. In this study, the texture of the newly produced analogs containing up to 60% hemp protein was comparable to that of the SPI product but the hemp protein concentrate required a higher denaturation temperature and absorbed less water. Moreover, physicochemical properties of hemp seed meal isolates were evaluated (i.e., water or fat-binding capacity, protein solubility, emulsification, foaming, and gelation) with regard to their potential use as a novel food component [8,20,21].

Hemp cake is obtained by squeezing the oil from seeds in cold-pressed oil production. When the oil is extracted with organic solvents from roasted seeds, this type of by-product is called a post-extraction meal. Hemp cake, as a by-product of cold pressing oil from hemp seeds, is a less processed ingredient, rich in protein, fiber, minerals, and biologically active compounds [7,11], and may be used for the production of new or reformulated meat products that will be perceived by consumers as healthier. Such products can also be perceived by consumers as more “environmentally friendly”, as they contain less animal raw materials and allow the use of valuable by-products for purposes other than the production of feed or waste. The present study aimed to assess the effect of the addition of hemp cake on the physicochemical, textural properties, protein and lipid oxidation, and sensory analysis of meatballs, cooked, vacuum-packed, and stored under refrigeration and to evaluate its application as a potential high-protein functional ingredient and an antioxidant additive.

## 2. Results

### 2.1. Changes in the Composition, and Physicochemical and Textural Properties

The percentage of the hemp additive in the final meatballs was in the range from 0.9% to 7.4% (Table 1). A control (M0) was a meatball sample without added HC. The proximate composition of the five prepared meatball treatments and the hemp cake (HC) added is shown in Table 2. The addition of 4.2% and 7.4% HC resulted in a significantly increased amount of dry matter and reduced the moisture content. Whereas no statically significant differences were observed in the protein and fat content (*p* > 0.05). HC contained only 1.51% fat and was rich in protein (31.47%) but the level of HC in the analysed meatballs turned out to be too low to significantly increase the protein content. Similarly, in a previously published study, the substitution of the 10% of minced beef for hemp flour in beef cutlets reduced water content, increased fat and mineral content but protein level was not considerably affected [18]. In gluten-free dough when a portion of the starch (10% or 20%) was replaced with hemp protein or hemp flour; the addition of 10% hemp increased the protein, fat, ash, and dietary fiber content [17].

The physicochemical properties of the cooked and vacuum-packed meatballs measured on days 1, 6 and 12 of storage are presented in Table 3. The pH values of the analysed samples ranged from 6.38 to 6.59. Regardless of the amount of hemp additive, pH remained virtually unchanged during the storage period. The pH values of raw meatballs ranged from 6.13 to 6.19 (Table S1 in Supplementary Material). Therefore, after cooking, the pH of the analysed samples increased. In a study aimed at examination of vegetable protein addition on cow’s yogurt quality, yogurt with natural hemp protein addition had only a slightly lower pH value than the control sample, i.e., 4.52 vs. 4.58 [16].

**Figure 1:**
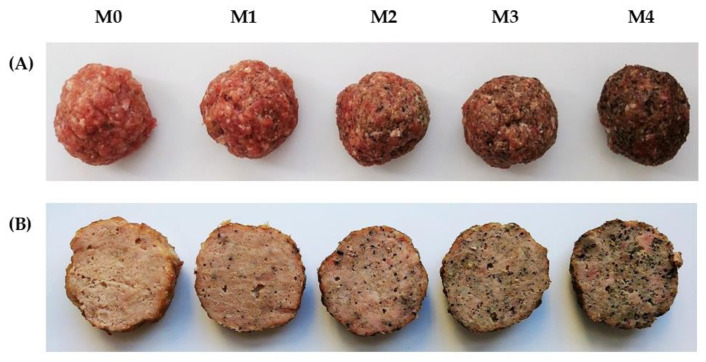
The meatballs before (**A**) and after (**B**) cooking. M0 = control; M1, M2, M3, M4 = meatballs with increasing content of hemp cake, i.e., 0.9%, 2.6%, 4.2% and 7.4%, respectively.

In relation to the colour parameters, considerable changes were measured between the five treatments. The hemp cake powder was dark with a greenish-brown tint, therefore the control meatballs were lighter. The photo of the meatballs before and after heat treatment is shown in Figure 1. The addition of HC created a “spicy/herbal look” on the surface and cross-section. Lightness (L*) and redness (a*) values reduced significantly with higher levels of hemp supplementation whereas yellowness (b*) was less affected (Table 3). There were no conclusive changes in colour during storage. The results are consistent with previously published findings where other hemp ingredients (hemp flour, protein, seeds) reduced the L* and a* values in meatloaves but b* was also less affected [9], whereas the substitution of soy protein isolate by hemp protein concentrate in meat analogues decreased the L* and b* values of products increased the a* parameter [19].

In meatballs with the greatest addition of HC (sample M4), significantly lower values of water activity were determined only between M1 and M4 treatments. Contrary to expectations, hemp cake had no considerable effect on reducing water loss despite its high protein content (31.47%). Cooking and storage losses decreased as the percentage of hemp increased though the differences were significant only between M0 and M3 variants for day 1 and day 6 (Table 3). In relation to storage time, storage losses were significantly higher (*p* < 0.05) in the examined samples after 12 days of storage and ranged from 4.19% to 3.25% for M0–M3. No significance in cooking and storage losses may result from poor hemp protein solubility at pH 4–6.5. As shown by Malomo et al. [20] research, defatted hemp protein meal had only around 13% solubility at pH 6 and even lower value (10%) at pH below 5.5, and its solubility increased only to around 25% at pH 9.0. The low protein solubility was related to the aggregation of hemp globulin proteins at pH below 7.0 and other protein-protein interactions during seed [20,22]. Therefore, by raising the pH and gentle processing, the water-holding capacity of the meatballs could be improved. Previously, Zając et al. [9] reported that the cooking losses were comparable between meatloaves with the addition of hemp flour, protein, and seeds; with no significant change compared to the control sample. Overall, the present research shows that the addition of hemp had no significant effect on pH changes and cooking losses in the examined meatballs.

The results of texture analyses are shown in Table 4. Some TPA parameters were significantly affected by the amount of the hemp ingredient and storage time. After 6 and 12 days of storage, the hardness of the hemp meatballs increased significantly compared to the M0 sample on the first day of storage. In the case of gumminess and chewiness, significantly higher values were found for the M1 and M2 variants after 12 days of storage compared to the control sample on the first day of storage. Those parameters are positively correlated with water-binding components such as protein and dietary fiber. With a longer storage time, the meatballs became harder, gummier, and chewier, regardless of the percentage of HC added. Whereas, springiness, cohesiveness, and resilience were not affected by either HC enrichment or storage period. The changes in texture profile parameters are dependent mainly on the amounts and interactions between all components in the mixture. Previous studies that described different effects of additives on the texture of meat products have been published so far, for instance, beef minces containing 15% of hemp protein exhibited improved stability and uniformity of the meat emulsions [6]. The addition of walnut green husk enhanced the hardness of cooked sausages while it lowered springiness and chewiness values [23]. Chickpea protein concentrate decreased the hardness, adhesivity, and chewiness of cooked “Merguez” sausage which was made from fresh beef and several herbal spices; and hemp ingredients (flour, protein, seeds) increased the hardness of meatloaves whilst other textural parameters were not affected [9,24].

**Figure 2:**
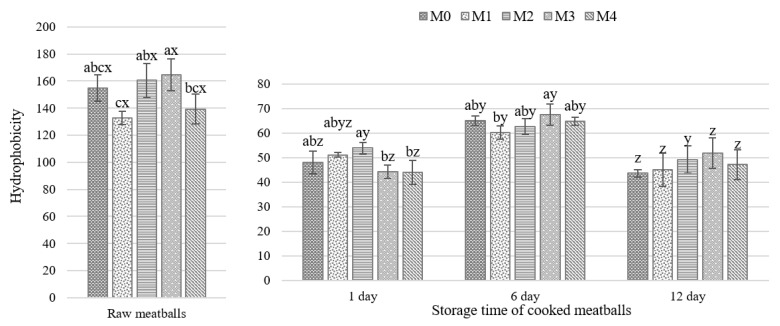
Changes in hydrophobicity of raw meatballs and during refrigerated storage of cooked meatballs with different hemp cake content; mean ± SD. a,b,c – different letters indicate a significant effect of hemp cake addition (*p* < 0.05); x,y,z – different letters indicate a significant effect of storage time (*p* < 0.05). M0 = control; M1, M2, M3, M4 = meatballs with increasing content of hemp cake, i.e., 0.9%, 2.6%, 4.2% and 7.4%, respectively.

The effects of cooking and HC addition on the surface hydrophobicity of proteins extracted using rigor buffer are shown in Figure 2. The cooked samples showed up to a three-fold reduction in hydrophobicity compared to raw samples but no conclusive relations were observed due to the hemp percentage added. Considering the storage time of cooked samples, hydrophobicity increased (*p* < 0.05) on the sixth day of refrigerated storage and then the values dropped on day 12. A declining trend in surface hydrophobicity of sarcoplasmic and myofibrillar proteins was reported previously during the storage of dried minced pork slices [25]. Although the statistical analysis indicates significant differences between the treatments analyzed (Figure 2 and Table S2 in Supplementary Material), it can be concluded that there is no apparent effect of the HC additive on protein surface hydrophobicity in the examined meatballs during refrigerated storage. It is likely that the hydrophobicity changes were affected by the extent of aggregation and polymerization processes occurring during heat treatment [26,27]. In our research, a correlation coefficient of 0.60 between the protein surface hydrophobicity of cooked meatballs and protein oxidation values indicated a moderate relationship although it was statistically significant (*p* < 0.05). Whereas the statistically significant correlation coefficient of 0.79 between the hydrophobicity and TBARS values represented a good association. Previously, lower surface hydrophobicity in the fermented sausage has been associated with inhibited protein oxidation and flavour development [28]. In general, physicochemical analysis, textural properties, and their changes during the storage period indicate that meatballs supplemented with hemp cake show no negative characteristics during storage up to 12 days.

### 2.2. Protein and Lipid Oxidation

**Figure 3:**
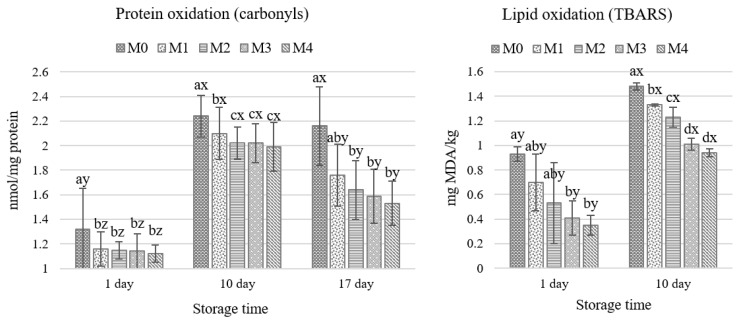
Effect of the addition of hemp cake and storage time on protein and lipid oxidation in cooked vacuum-packed meatballs; mean ± SD. a,b,c – different letters indicate a significant effect of hemp cake addition (*p* < 0.05); x,y,z – different letters indicate a significant effect of storage time (*p* < 0.05). M0 = control; M1, M2, M3, M4 = meatballs with increasing content of hemp cake, i.e., 0.9%, 2.6%, 4.2% and 7.4%, respectively.

The addition of HC affected the level of protein and lipid oxidation during refrigerated storage (Figure 3). Since products of protein and lipid oxidation lead to the production of end products that worsen sensory quality, oxidation reactions were followed for 17 days. The values of protein oxidation in the meatballs expressed as carbonyl content (nmol carbonyls per mg protein) were considerably higher after 10 and 17 days of storage. The increase was significantly higher in the control samples (M0) than in the four treated variants. The addition of HC significantly inhibited the formation of protein carbonyls in the meatballs, e.g., on day 10 carbonyls ranged from 2.24 nmol/mg protein in M0 to 1.99 nmol/mg protein in M4; the inhibition effect ranged from 6.25% in M1 to 11.16% in M4. Reduced carbonyl content after prolonged storage (17 days) was measured in all exam-ined samples. Turgut, Soyer, and Işıkçı [29] depicted a similar rise and fall in carbonyl levels in all raw beef meatballs, including control samples and those enriched with pom-egranate peel extract, during refrigerated storage. Therefore, the present results indicate the potential antioxidant activity of HC and its active protective effect during meatball storage. Various studies have shown that cooked muscle proteins are susceptible to an increase in the formation of carbonyl compounds which is caused by the oxidative degradation of side chains of arginine, histidine, lysine, and proline residues [30].

There are no published studies in the scientific literature on the changes in carbonyls in food products affected by the addition of hemp ingredients. However, several studies performed in vitro have demonstrated that hemp seed proteins release bioactive peptides with antioxidant activity. For example, when hemp seed protein isolate was subjected to simulated gastrointestinal tract digestion with pepsin-pancreatin hydrolysis, the proce-dure revealed short-chain peptides (≤5 amino acids) having DPPH radical scavenging and metal chelation activity up to 67% and 96%, respectively [31]. However, Lin et al. [12] reported that preheating of isolated hemp protein lowered DPPH scavenging activities of antioxidative peptides compared to unheated pepsin-pancreatin hydrolysates, though heat pretreatment improved the digestibility of hemp protein due to the enhancement of low molecular weight proteins and degree of hydrolysis [12]. In the present study, the increase in carbonyl content was significantly higher in the M0 than in the four treated samples. Therefore, the antioxidant activity may be related to the non-hydrolyzed hemp protein that was present in hemp cake. However, further research is needed to confirm this phenomenon. Increases in carbonyl concentration as well as the antioxidant activity of fruit extracts (3% of total weight) against protein carbonyl formation have been reported in emulsified cooked porcine burger patties [32]. It should be emphasized that in the present study on the influence of hemp cake, smaller amounts of protein carbonyls were detected, likely, among other reasons, as a result of different packaging methods, i.e., we used vacuum packaging in barrier bags whereas burger patties were wrapped with PVC film.

An increase in lipid oxidation was detected in all samples after 10 days of storage but hemp cake treatments exhibited protective properties against lipid oxidation when com-pared to the M0 (Figure 3). Significantly lower TBARS values were found in meatballs M3 and M4 on the 1st and 10th days of storage; the inhibitory effect in the M4 variant reached 62.4% and 36.5% in both terms, respectively, compared to the control, M0 (Table S3 in Supplementary Material). The TBARS values measured on the 17th day showed high var-iability and were therefore excluded from this study. Moreover, in the present study, a correlation coefficient of 0.91 between TBARS and carbonyl content indicated a very strong relationship (*p* < 0.05). The antioxidant activity observed in the meatballs may be related to the antioxidant compounds present (e.g., polyphenols) in the HC. A previous study showed that phenols are mainly accumulated in the seed hull since much higher total phenolic content was determined for defatted hemp seed hull than seed kernel (e.g., 0.92–9.60 mg GAE/100 mg extract versus 0.79–1.56 mg GAE/100 mg extract for Yunma variety hull and kernel, respectively, depending on the type of extraction) [33]. While lower total phenolic content (0.44 mg GE/g,) was observed in the cold-pressed hemp seed oil extract [34]. This was confirmed by Zając et al. (2019) who detected a significant decrease in oxidation processes for pork loaves prepared with ingredients containing hemp hull (hemp flour, protein, and seeds) [9]. The present results show that HC can be a potentially effective natural antioxidant source to inhibit processes of protein and lipid oxidation in meatballs during refrigerated storage, especially if it is in a concentration above 3-5% in the product.

### 2.3. Sensory Evaluation

**Figure 4:**
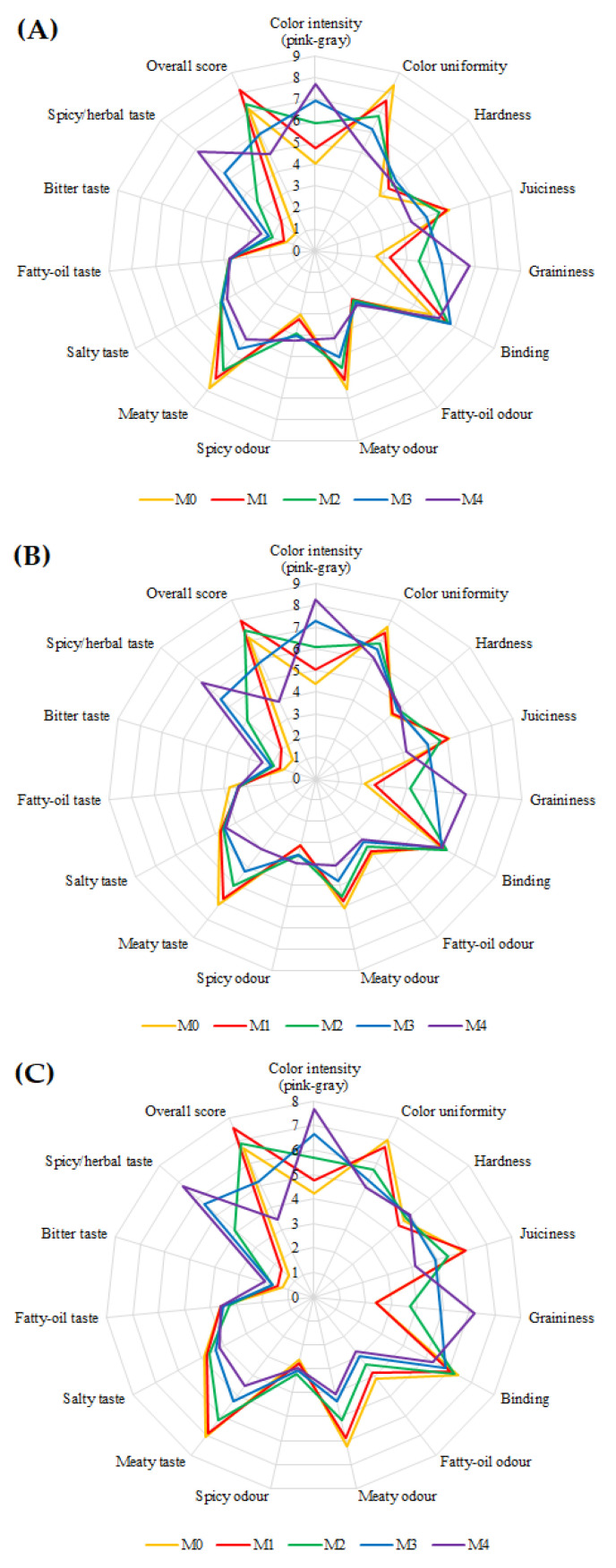
The spider chart represents mean sensory analysis scores for the five treatments of cooked and vacuum-packed meatballs evaluated on day 1st (**A**), 6th (**B**), and 12th (**C**) during refrigerated storage.

The results from the sensory evaluation are shown in Figure 4. Regarding the influence of HC addition, texture parameters, such as hardness and binding were not statistically different between the examined treatments. However, the visible differences in appearance (Figure 1) were reflected in other sensory descriptors. Colour intensity, graininess, and spicy/herbal taste were graded significantly higher for meatballs containing hemp cake compared to the control sample. Whereas colour uniformity, juiciness, meaty odour, and meaty taste were evaluated inversely with the hemp content. After 1, 6, and 12 days of storage, the product M4 with the highest HC content (7.4%) obtained the lowest overall score and it was significantly lower compared to the M0, M1, and M2. The highest overall scores were obtained for M1 prepared with 0.9% hemp cake. In each of the analyzed storage times, the M1 samples were significantly higher-rated compared to M3 and M4 (Figure 4A; Table S4). On their assessment cards, the respondents declared the sensation of poppy seeds and sweet/herbal taste in each treatment of meatballs enriched with HC. These features likely contributed to their positive assessment. Greenish, spicy in appearance HC content of up to 2.6% resembled typical herbs/spices and did not negatively affect the sensory evaluation (Figure 1). When the influence of storage time on the sensory properties of cooked meatballs was tested, only data for colour uniformity, fatty-oil odour, and meaty taste were significant, as well as the overall scores which decreased after 12 days of storage for all the examined treatments (Figure 4B,C; Tables S4 and S5). The sensory evaluation results show that the threshold acceptance for cooked meatballs was the concentration of 2.6% HC since higher contents were less acceptable for panelists. The results of two-way ANOVA showed that among parameters that significantly affected and reduced the overall score were colour, juiciness, graininess, meaty odour, and bitter taste (Table S5). Previously reported the overall scores of the quality of beef cutlets were reduced with the increasing doses of hemp flour (5, 10, and 15%). Although a significant change in color and taste was obtained when 10% of beef was substituted with 10% of hemp flour, this product was assessed as still maintaining acceptable flavour characteristics [18]. The meatballs’ acceptability was likely more susceptible to significantly decrease, even though a lower amount of a hemp product was added in comparison with beef cutlets. The reason may be a less pronounced pork flavor and colour compared to beef and the addition of raw fat in the beef cutlet recipe, which could increase the taste and aroma of the beef cutlets.

The present results confirm the conclusions of other scientists that a moderate amount of hemp ingredient in food products is a desirable feature. In a study on energy bars made from extruded rice and whole hemp powder, bars with the highest hemp share (40%) had the lowest overall acceptability, while those with the lowest (20%) were the preferred product [35]. Therefore, on the one hand, a negative correlation was determined between the addition of hemp flour to wheat bread and gluten-free biscuits and the results of the sensory evaluation [36,37]. The reasons for the negative reception of those products were a bitter aftertaste, hay-like aroma, and an undesirable dark green colour. On the other hand, gluten-free crackers made from brown rice flour and up to 20% hemp cake were evaluated as tasty and crunchy, with a pleasant nutty flavour [38]. Zając et al. [9] reported that although 5% of hemp ingredients lowered the overall acceptability of pork loaves, consumers would be willing to buy these products if the information on their health-promoting properties were provided on the label.

In summary, the results of the present study suggest that hemp cake has potential applicability in meat processing as a sustainable and antioxidant ingredient. However, further research is necessary to understand the impact of hemp cake on nutrient digestibility and undesirable reactions to food, for instance, food intolerances because of the risk that meat products containing ingredients from new sources may cause undesirable gastrointestinal symptoms. Therefore, more research in these directions is warranted.

## 3. Materials and Methods

### 3.1. Preparation of Meatballs

Hemp seeds (*Cannabis sativa* L.) of the Finola variety were obtained from the Polish company SemCo Sp. z o.o. (Szamotuły near Poznań, Poland). The hemp cake was prepared by the cold pressing process using a Yoda oil press YD-ZY-02A (Warsaw, Poland) as described previously [39]. The resulting cake was dried overnight in a drying oven at 40 °C and then ground in a grinder (Bosch GmbH, Gerlingen-Schillerhöhe, Germany). The meatball formulation was based upon a typical meatball recipe, which is a popular type of product consumed in Poland. The pork meat was purchased at a local store; rabbit and guinea fowl meats were purchased at Makro Cash and Carry hypermarket. The meats were stripped of bones and skin and then ground separately using a Zelmer type 685.5 electric mincer (Zelmer S.A., Rzeszów, Poland) through a 3 mm mesh. Subsequently, the minced meat was divided into five equal portions and mixed with the same amount of water, bread crumbs, salt, and spices, and finally, hemp cake (HC) was added into each portion based on total meat weight percentage, obtaining the experimental treatments M1-M4, respectively. The final composition of the meatball treatments is shown in Table 1.

The batter was mixed manually until the ingredients were spread evenly. The batter was left for approximately 30 min before the 50 ± 1 g meatballs were formed. Each treatment was placed on a stainless steel baking tray and cooked in a Rational Combi model SCC 61 convection oven (Landsberg am Lech, Germany). Heating was carried out at a temperature of 160 °C with an air humidity in the oven chamber of 75% until reaching a core temperature of 72 °C. After cooling, the meatballs were vacuum-packed in barrier bags using a Multivac C100 chamber machine and stored at 5 ± 1 °C for 17 days. Samples were analysed on the 1st, 6th, and 12th days of storage; protein and lipid oxidation was measured on the 1st, 10th, and 17th days of storage. The experiment was replicated twice and assays were performed in triplicate per treatment and per storage time.

### 3.2. Proximate Composition

The proximate composition was determined on cooked meatballs on the 1st day of storage, in triplicate. Samples for physicochemical analysis were homogenized using a Moulinex S 643.23 food processor. Protein (Kjeldahl method), water/moisture, fat (Soxhlet method), and ash were evaluated according to ISO standards PN-EN ISO 8968-1:2014-03, PN-ISO 1442:2000, PN-ISO 1444:2000, and PN-ISO 936:2000, respectively.

### 3.3. pH, Color, and Water Activity Measurements

The pH and colour values were determined on raw samples (0 days of storage) and cooked, vacuum-packed meatballs stored for 1, 6, 12 days. The pH was measured using a portable Handylab 2 m (Schott AG, Mainz, Germany) equipped with a Schott L68880 pH combination electrode (PN-ISO 2917:2001). Before measurements, the pH meter was calibrated at room temperature using pH buffers 4.01 and 7.00 (Testo Ltd., Alton Hampshire, UK). Measurements were taken at room temperature (22–24 °C). Colour (CIE L*, a*, b*) was measured in triplicate on the cross-section of freshly cut meatballs using a Dr. Lange LMG161 portable colorimeter (Dr. Bruno Lange GmbH and Co. KG, Berlin, Germany). The parameters were set to illuminant D65, observer angle of 10°, the aperture size of 5.0 mm. Before measuring the colorimeter was calibrated against a white standard (no. 3125). Water activity of cooked meatballs was measured using a LabMaster-aw neo instrument (Novasina AG, Lachen, Switzerland) at 25 ± 1 °C. Before measurements, the meter was calibrated with SAL-T 97% calibration standard with automatic recognition by RFID tag.

### 3.4. Cooking and Storage Losses

The cooking losses were determined after the samples had cooled down at room temperature. The cooking and storage losses were calculated by weighing the samples before and after thermal treatment or the storage period, respectively. The values are presented as the percentage of the mass noted before a given treatment. The storage losses were measured after 1, 6, and 12 days of storage.

### 3.5. Texture Profile Analysis

Texture profile analysis (TPA) was carried out using a TA.XT.plus texture analyser (Stable Micro Systems, UK) at room temperature. Cooked and cooled samples were cut into a cylindrical shape (diameter of 14 mm and a length of 15 mm). The measurements were conducted with the following settings: probe travel rate before testing 5 mm/s, during and after testing 2 mm/s, strain 50%, and a 3 s interval between the first and second stroke. Eight (sixteen in total) measurements were performed for each sample treatment and storage period. The following texture profile parameters were determined: Hardness, springiness, cohesiveness, gumminess, chewiness, and resilience.

### 3.6. Protein Surface Hydrophobicity

Protein surface hydrophobicity for raw and cooked meatballs was determined according to Li-Chan, Nakai, and Wood [40] based on fluorescence measurements using ANS reagent (8-anilino-1-naphthalenesulfonic acid) that reacts with residues of aromatic amino acids (phenylalanine, tyrosine, tryptophan); thus, this assay is frequently referred to as aromatic hydrophobicity. Protein was extracted using rigor buffer (pH 7.0) from raw (0 days) and cooked meatballs, the latter after 1, 6, and 12 days of [41]. The protein concentration was determined using the Biuret method. Relative intensity of fluorescence at the wavelengths of 390 nm (excitation) and 490 nm (emission) was measured in samples with known protein concentrations using an LS-55 luminescence spectrometer (PerkinElmer Inc., Waltham, MA, USA.). Surface hydrophobicity (So) constituted the directional coefficient of the determined straight line.

### 3.7. Protein Oxidation (Carbonyl Content)

Protein oxidation was measured by the total carbonyl content by derivatization with 2,4-dinitrophenyl hydrazine (DNPH) according to Levine, Wehr, Williams, Stadtman, and Shacter [42] with slight modifications. Cooked meatballs (1 g) were homogenized 1:10 (*w*/*v*) in 20 mM sodium phosphate buffer containing 6 M NaCl (pH 6.5) using an Ultra Turrax homogenizer for 30 s. The homogenate was filtered through a filter paper. Two aliquots of 0.4 mL were taken from the homogenate and dispensed in 2 mL Eppendorf tubes. Proteins were precipitated by cold 10% TCA (1.5 mL), incubated at 20 °C for 20 min and subsequently centrifuged for 5 min at 4 °C at 10,000× *g*. The supernatant was discarded and one pellet was treated with 1 mL of 2 M HCl for protein concentration measurement and the other with 1 mL of 10 M DNPH in 2 M HCl for carbonyl concentration measurement. Both samples were incubated for 30 min at 37 °C. Afterward, samples were precipitated by 1 mL of 10% TCA, centrifuged for 5 min at 4 °C at 10,000× *g*, and washed three times with 1 mL of ethanol:ethyl acetate (1:1, *v*/*v*) to remove excess DNPH. The pellets were then dried for 1 h at room temperature. Subsequently, the pellets were dissolved in 1.5 mL of 20 mM sodium phosphate buffer containing 6 M guanidine hydrochloride (pH 6.5), incubated for 30 min at 37 °C and centrifuged for 5 min at 4 °C at 10,000× *g* to remove insoluble fragments. The carbonyl concentration (nmol/mg protein) was calculated from the absorbance of the samples at 280 and 370 nm using the following equation [42]:

$$\frac{c\;hydrazone}{c\;protein}=\frac{A_{370}}{22,000\times \left(A_{280}-A_{370}\times 0.43\right)}\times 10^{6}.$$

### 3.8. Lipid Oxidation

Lipid oxidation was determined by measuring thiobarbituric acid-reactive substances (TBARS) as described by Doolaege et al. [34] with slight modifications. Cooked meatball samples (5 g) were homogenized in 20 mL of 0.6 M HClO_4_ and 1 mL butylated hydroxytoluene (BHT) solution using an Ultra Turrax for 30 s. Then, another 20 mL of HClO_4_ and BHT solution was added and the mixture was shaken for 30 min. The homogenate was filtered, 5 mL was transferred to a glass test-tube, and 1 mL of TBA reagent was added. The resulting solution was heated in a thermoblock at 100 °C for 35 min. The samples were subsequently cooled to room temperature, centrifuged (5 min, 10,000× *g*, 20 °C), and the absorbance was measured at 532 nm. TBARS values were calculated using a standard curve and expressed as mg malondialdehyde equivalents (MDA eq.)/kg sample.

### 3.9. Sensory Evaluation

An eight-membered panel group evaluated the meatballs on days 1, 6, and 12 during refrigerated storage. The panelists (25–62 years, 8 females) were experienced in the sensory evaluation of food, specific training associated with these samples was not considered. Sensory evaluation was conducted according to a previous protocol [43] with some modifications. The samples were evaluated in a bright space at room temperature. Five coded products were given to each person on a plastic plate. The products were evaluated based on a graphical, unstructured scale, using a continuous 10 cm line scale with the endpoint descriptors from “none” to “very intense”. The following attributes were assessed: colour from pink to gray (intensity, uniformity), texture (hardness, juiciness, graininess, binding), odour (fatty-oil, meaty-cooked meat, spicy/herbal, rancid), taste (meaty-cooked meat/umami, salty, fatty-oil, bitter, spicy/herbal), and overall assessment (from extremely distasteful to extremely good).

### 3.10. Statistical Analysis

Data analysis was performed using Statistica 13.1 (StatSoft Inc., Tulsa, OK, USA.) to evaluate the effect of treatment (M0-M4) and storage period for meatballs in three terms (physicochemical properties, texture parameters sensory analysis on days 1, 6, and 12; oxidation on days 1, 10, and 17). Analysis of variance (ANOVA) and Tukey’s test of multiple comparisons were used to determine significant differences between mean values. Additionally, the Pearson correlation coefficient (r) was computed, which measures a linear correlation/dependence between two variables. The results were considered to be statistically significant at *p* < 0.05. The results were expressed as mean ± standard deviation. The resulting values were imported into MS Excel 2019 (Microsoft, Redmond, WA, USA) for figure generation.

## 4. Conclusions

Meatballs were enriched with different percentages of hemp cake, a by-product of the cold pressing process, which is a valuable nutritious ingredient. This study demonstrated that the addition of HC affected some of the physicochemical, oxidative, and sensory properties of meatballs. The addition of hemp cake reduced protein carbonylation and TBARS values, which may result in slower protein and lipid oxidation processes related to the negative changes in the technological and sensory properties of the final product during refrigerated storage. Overall scores for the sensory evaluation of samples prepared with 0.9% and 2.6% of HC were higher than that of the control but lower overall scores were found for samples containing 4.2% and 7.4% HC. In general, physicochemical analysis, textural properties, and their changes during the storage period indicate that meatballs supplemented with hemp cake show no visible signs of deterioration, and can be kept refrigerated for up to 12 days. It is highly probable that vacuum packing has slowed down the growth of mesophilic aerobic bacteria but the future direction is to perform extend microbiological analyses to assess microbiological quality during shelf life. In conclusion, the present results confirm that hemp cake may be applied in meat processing as a functional ingredient provided that the level tolerated by consumers is maintained.

## Figures and Tables

**Table 1:** Percentage composition of the five variants of meatballs.

Ingredients	Meatballs				
	M0	M1	M2	M3	M4
Pork shoulder	66.0	65.4	64.3	63.2	61.1
Rabbit meat	10.9	10.8	10.6	10.4	10.1
Guinea fowl meat	7.3	7.2	7.1	7.0	6.7
Water	8.9	8.8	8.6	8.5	8.2
Bread crumbs	5.3	5.3	5.2	5.1	5.0
Salt	1.3	1.3	1.3	1.3	1.2
Pepper	0.2	0.2	0.2	0.2	0.2
Garlic	0.1	0.1	0.1	0.1	0.1
Hemp cake	0.0	0.9	2.6	4.2	7.4

**Table 2:** Proximate composition of cooked meatballs and hemp cake; mean ± SD.

Parameter	Meatballs					Hemp Cake HC
	M0	M1	M2	M3	M4	
Protein	18.04 ± 0.84	18.33 ± 0.46	18.36 ± 0.72	18.70 ± 0.37	19.40 ± 1.00	31.47 ± 0.23
Moisture	67.88 ± 0.57 ^a^	68.01 ± 0.85 ^a^	67.37 ± 0.67 ^a,b^	65.86 ± 0.71 ^b^	63.57 ± 1.57 ^c^	9.84 ± 0.17
Fat	6.82 ± 0.13	6.98 ± 0.08	7.84 ± 1.45	7.78 ± 0.93	7.41 ± 1.36	1.51 ± 0.20
Dry matter	25.30 ± 0.60 ^b,c^	25.01 ± 0.92 ^c^	24.79 ± 0.99 ^c^	26.36 ± 0.33 ^b^	29.02 ± 0.07 ^a^	88.65 ± 0.08
Ash	2.20 ± 0.07	2.13 ± 0.06	2.30 ± 0.24	2.11 ± 0.19	2.35 ± 0.12	7.05 ± 0.08

^a,b,c^ – Means with different letters in a row indicate a significant effect of hemp cake addition (*p* < 0.05).M0 = control; M1, M2, M3, M4 = meatballs with increasing content of hemp cake, i.e., 0.9%, 2.6%, 4.2% and 7.4%, respectively.

**Table 3:** Physicochemical properties of cooked meatballs measured during storage; mean ± SD.

Parameter	Day	Meatballs				
		M0	M1	M2	M3	M4
pH	1	6.48 ± 0.08 ^a,b^	6.43 ± 0.03 ^a,b^	6.51 ± 0.03 ^a^	6.40 ± 0.02 ^a,b^	6.36 ± 0.10 ^b^
	6	6.51 ± 0.03 ^a,b^	6.46 ± 0.05 ^b^	6.59 ± 0.03 ^a^	6.50 ± 0.1 ^a,b^	6.41 ± 0.04 ^b^
	12	6.38 ± 0.05 ^b^	6.40 ± 0.05 ^b^	6.55 ± 0.07 ^a^	6.48 ± 0.08 ^a,b^	6.41 ± 0.06 ^b^
*L*	1	65.52 ± 1.36 ^a^	61.73 ± 1.11 ^b^	57.72 ± 2.19 ^c^	58.06 ± 0.48 ^c,x^	52.76 ± 1.17 ^d^
	6	64.25 ± 1.09 ^a^	62.50 ± 0.26 ^b^	56.02 ± 0.71 ^c^	56.00 ± 0.42 ^c,y^	53.00 ± 0.91 ^d^
	12	63.95 ± 0.75 ^a^	60.83 ± 0.40 ^b^	59.43 ± 0.85 ^b,c^	58.20 ± 1.62 ^c,x^	54.80 ± 0.9 ^d^
a*	1	5.14 ± 0.38 ^a,x^	4.43 ± 0.24 ^b^	4.00 ± 0.23 ^b^	3.96 ± 0.27 ^b,x^	3.22 ± 0.29 ^c,x^
	6	4.33 ± 0.35 ^a,y^	4.28 ± 0.13 ^a^	3.60 ± 0.24 ^b^	2.83 ± 0.17 ^b,y^	2.50 ± 0.32 ^c,y^
	12	4.58 ± 0.22^a,y^	4.25 ± 0.26 ^a,b^	4.00 ± 0.52 ^a,b^	3.84 ± 0.32 ^b,x^	2.54 ± 0.32 ^c,y^
b*	1	11.40 ± 0.30 ^a,b^	12.48 ± 0.67 ^a,x^	9.64 ± 0.86 ^c,x,y^	10.36 ± 0.97 ^b,c^	11.26 ± 0.98 ^a,b^
	6	11.28 ± 0.45 ^a^	11.85 ± 0.30 ^a,x,y^	8.10 ± 0.47 ^c,y^	10.23 ± 0.63 ^b^	10.90 ± 0.48 ^a,b^
	12	11.88 ± 0.46 ^a^	11.43 ± 0.25 ^a,b,y^	10.25 ± 0.48 ^b,c,x^	9.76 ± 0.99 ^c^	9.92 ± 0.66 ^c^
Water activity	1	0.9792 ± 0.0033 ^a,b^	0.9803 ± 0.0014 ^a^	0.9788 ± 0.0007 ^a,b^	0.9779 ± 0.0015 ^a,b^	0.9770 ± 0.0010 ^b^
Cooking loss [%]	0	12.72 ± 1.17	12.04 ± 0.86	11.64 ± 0.65	11.13 ± 1.07	10.71 ± 1.08
Storage loss [%]	1	2.19 ± 0.38 ^a,z^	1.74 ± 0.25 ^a,b,z^	1.84 ± 0.48 ^a,b,z^	1.36 ± 0.48 ^b,z^	1.6 ± 0.39 ^a,b,y^
	6	3.24 ± 0.55 ^a,y^	2.76 ± 0.36 ^a,b,y^	2.91 ± 0.39 ^a,b,y^	2.29 ± 0.41 ^b,y^	2.42 ± 0.75 ^a,b,y^
	12	4.19 ± 0.76 ^x^	3.7 ± 0.48 ^x^	3.88 ± 0.65 ^x^	3.25 ± 0.46 ^x^	3.41 ± 0.60 ^x^

^a,b,c^ – Means with different letters in a row indicate a significant effect of hemp cake addition (one-way ANOVA and post hoc Tukey test; *p* < 0.05); ^x,y,z^ – means with different letters in a column for particular parameters indicate a significant effect of storage time (one-way ANOVA and post hoc Tukey test; *p* < 0.05).

**Table 4:** Texture profile analysis of the five variants of cooked meatballs stored in refrigerated conditions; mean ± SD.

Parameter	Day	Meatballs				
		M0	M1	M2	M3	M4
Hardness [N]	1	9.70 ± 1.69 ^c^	11.16 ± 2.71 ^a,b,c^	11.21 ± 1.12 ^a,b,c^	11.00 ± 1.72 ^b,c^	11.15 ± 2.50 ^a,b,c^
	6	12.84 ± 1.96 ^a,b^	13.11 ± 3.35 ^a,b^	13.72 ± 1.64 ^a,b^	12.80 ± 1.48 ^a,b^	13.14 ± 2.87 ^a,b^
	12	11.80 ± 1.86 ^a,b,c^	13.21 ± 3.25 ^a,b^	13.96 ± 1.22 ^a^	13.34 ± 1.1 ^a,b^	13.68 ± 2.52 ^a,b^
Springiness	1	0.67 ± 0.04 ^a,b^	0.65 ± 0.09 ^a,b^	0.66 ± 0.04 ^a,b^	0.65 ± 0.05 ^a,b^	0.62 ± 0.08 ^b^
	6	0.70 ± 0.04 ^a,b^	0.72 ± 0.06 ^a^	0.69 ± 0.05 ^a,b^	0.66 ± 0.04 ^a,b^	0.65 ± 0.07 ^a,b^
	12	0.66 ± 0.09 ^a,b^	0.72 ± 0.04 ^a^	0.71 ± 0.04 ^a^	0.66 ± 0.04 ^a,b^	0.66 ± 0.07 ^a,b^
Cohesiveness	1	0.39 ± 0.05	0.41 ± 0.09	0.40 ± 0.06	0.39 ± 0.07	0.38 ± 0.07
	6	0.39 ± 0.05	0.39 ± 0.06	0.42 ± 0.05	0.40 ± 0.04	0.37 ± 0.07
	12	0.38 ± 0.06	0.43 ± 0.07	0.43 ± 0.05	0.40 ± 0.04	0.38 ± 0.06
Gumminess	1	3.87 ± 1.03 ^b^	4.8 ± 2.02 ^a,b^	4.5 ± 1.17 ^a,b^	4.41 ± 1.4 ^a,b^	4.34 ± 1.57 ^a,b^
	6	5.08 ± 1.28 ^a,b^	5.20 ± 2.05 ^a,b^	5.82 ± 1.31 ^a,b^	5.11 ± 1.06 ^a,b^	5.01 ± 1.80 ^a,b^
	12	4.53 ± 1.08 ^a,b^	5.89 ± 2.26 ^a^	6.06 ± 1.05 ^a^	5.41 ± 0.84 ^a,b^	5.36 ± 1.68 ^a,b^
Chewiness	1	2.62 ± 0.81 ^b^	3.23 ± 1.59 ^a,b^	2.97 ± 0.8 ^a,b^	2.85 ± 0.81 ^a,b^	2.76 ± 1.19 ^a,b^
	6	3.54 ± 0.91 ^a,b^	3.78 ± 1.54 ^a,b^	4.05 ± 1.08 ^a,b^	3.39 ± 0.87 ^a,b^	3.34 ± 1.44 ^a,b^
	12	3.07 ± 1.01 ^a,b^	4.24 ± 1.71 ^a^	4.31 ± 0.88 ^a^	3.58 ± 0.74 ^a,b^	3.63 ± 1.39 ^a,b^
Resilience	1	0.16 ± 0.03 ^a,b^	0.17 ± 0.05 ^a,b^	0.16 ± 0.03 ^a,b^	0.15 ± 0.04 ^a,b^	0.14 ± 0.04 ^a,b^
	6	0.16 ± 0.02 ^a,b^	0.16 ± 0.03 ^a,b^	0.17 ± 0.02 ^a,b^	0.15 ± 0.02 ^a,b^	0.13 ± 0.03 ^b^
	12	0.15 ± 0.03 ^a,b^	0.18 ± 0.04 ^a^	0.18 ± 0.03 ^a^	0.15 ± 0.02 ^a,b^	0.14 ± 0.03 ^a,b^

^a,b,c^ – Means with different letters for particular parameters in rows and columns indicate a significant effect (two-way ANOVA and *post hoc* Tukey test) (*p* < 0.05).

## Data Availability

The data supporting reported results are available from the corresponding author on reasonable request.

## Supplementary Materials

The following are available online, Table S1: pH and colour values of raw meatballs measured on 0 days of storage; Table S2: Changes in hydrophobicity of raw and cooked, vacuum-packed meatballs with different hemp cake content; Table S3: Protein and lipid oxidation values of cooked and vacuum-packed meatballs during storage.

## Sample Availability

Not available.
