# Supplementary material for: The Effect of Hemp Cake (Cannabis sativa L.) on the Characteristics of Meatballs Stored in Refrigerated Conditions
Source: Molecules. 2021 Aug 31;26(17):5284. doi: 10.3390/molecules26175284 (PMC8433655; doi:10.3390/molecules26175284)
Supplement: Supplementary file 1 [file molecules-26-05284-s001.zip › molecules-1318905-supplementary.pdf]

Supplementary Materials

The Effect of Hemp Cake (*Cannabis sativa* L.) on the Charac-teristics of Meatballs Stored in Refrigerated Conditions

Klaudia Kotecka-Majchrzak, Natalia Kasałka-Czarna, Anita Spychaj , Beata Mikołajczak and Magdalena Montowska *

Department of Meat Technology, Poznan University of Life Sciences, Wojska Polskiego 31, 60-624 Poznan, Poland; klaudia.kotecka@up.poznan.pl (K.K.-M.); natalia.czarna@up.poznan.pl (N.K.-C.); anita.spychaj@up.poznan.pl (A.S.); beata.mikolajczak@up.poznan.pl (B.M.)
* Correspondence: magdalena.montowska@up.poznan.pl; Tel.: +48-61-848-7257

Tables:

Table S1
pH and colour values of raw meatballs measured on 0 days of storage; mean ± SD.

Table S2
Changes in hydrophobicity of raw and cooked, vacuum-packed meatballs with different hemp cake content; mean ± SD.

Table S3
Protein and lipid oxidation values of cooked and vacuum-packed meatballs during storage; mean ± SD.

Table S4
Influence of hemp cake addition on the sensory properties of cooked and vacuum-packed meatballs during storage; mean ± SD.

Table S5
Influence of the storage time (T), hemp cake addition (H), and their interaction on the sensory analysis of meatballs.

Table S1

pH and colour values of raw meatballs measured on 0 days of storage; mean ± SD.

Parameter	Meatballs				
	**M0**	**M1**	**M2**	**M3**	**M4**
pH	6.13 ± 0.12	6.14 ± 0.10	6.19 ± 0.05	6.14 ± 0.06	6.19 ± 0.06
L	45.96 ± 2.29	46.88 ± 2.15	45.91 ± 1.76	45.53 ± 2.75	43.83 ± 2.15
*a**	3.24 ± 1.06	3.15 ± 0.66	3.95 ± 1.34	3.30 ± 1.27	2.68 ± 0.46
*b**	11.31 ± 0.90^{b}	12.20 ± 1.46^{ab}	13.55 ± 1.30^{a}	13.56 ± 1.43^{a}	13.29 ± 1.21^{a}

^{a,b,c} – Means with different letters in a row indicate a significant effect of hemp cake addition ($P < 0.05$).
M0 = control; M1, M2, M3, M4 = meatballs with increasing content of hemp cake, i.e., 0.9%, 2.6%, 4.2% and 7.4%, respectively.

Table S2

Changes in hydrophobicity of raw and cooked, vacuum-packed meatballs with different hemp cake content; mean ± SD.

Hydrophobicity	Meatballs				
	**M0**	**M1**	**M2**	**M3**	**M4**
Raw samples	154.81 ± 9.8^{abcx}	132.64 ± 4.89^{cx}	160.61 ± 12.56^{abx}	164.40 ± 11.8^{ax}	139.30 ± 10.92^{bcx}
Cooked samples					
1 day	48.12 ± 4.65^{abz}	51.15 ± 0.89^{abyz}	53.93 ± 2.32^{ay}	44.28 ± 2.77^{bz}	44.01 ± 4.90^{bz}
6 days	65.01 ± 1.89^{aby}	60.34 ± 2.70^{by}	62.71 ± 3.3^{aby}	67.50 ± 4.42^{ay}	64.79 ± 1.53^{aby}
12 days	43.67 ± 1.47^{z}	45.14 ± 6.75^{z}	49.27 ± 5.55^{y}	51.87 ± 6.20^{z}	47.17 ± 6.04^{z}

^{a,b,c} – Means with different letters in a row indicate a significant effect of hemp cake addition ($P < 0.05$);
^{x,y,z} – means with different letters in a column indicate a significant effect of storage time ($P < 0.05$).

Table S3

Protein and lipid oxidation values of cooked and vacuum-packed meatballs during storage; mean ± SD.

Parameter	Day	Meatballs				
		**M0**	**M1**	**M2**	**M3**	**M4**
Protein oxidation	1	1.32 ± 0.33^{ay}	1.16 ± 0.14^{bz}	1.15 ± 0.07^{bz}	1.14 ± 0.14^{bz}	1.12 ± 0.07^{bz}
	10	2.24 ± 0.17^{ax}	2.10 ± 0.21^{bx}	2.02 ± 0.13^{cx}	2.02 ± 0.16^{cx}	1.99 ± 0.20^{cx}
	17	2.16 ± 0.32^{ax}	1.76 ± 0.25^{aby}	1.64 ± 0.24^{by}	1.59 ± 0.22^{by}	1.53 ± 0.18^{by}
Lipid oxidation	1	0.93 ± 0.06^{ay}	0.70 ± 0.23^{aby}	0.53 ± 0.33^{aby}	0.41 ± 0.14^{by}	0.35 ± 0.08^{by}
	10	1.48 ± 0.03^{ax}	1.33 ± 0.01^{bx}	1.23 ± 0.08^{cx}	1.01 ± 0.05^{dx}	0.94 ± 0.03^{dx}

^{a,b,c} – Means with different letters in a row indicate a significant effect of hemp cake addition ($P < 0.05$);
^{x,y,z} – means with different letters for particular parameter in a column indicate a significant effect of storage time ($P < 0.05$).

Table S4
Influence of hemp cake addition on the sensory properties of cooked and vacuum-packed meatballs during storage; mean ± SD.

Parameter	Day	Meatballs				
		**M0**	**M1**	**M2**	**M3**	**M4**
Color intensity	1	$4.03^{c} \pm 1.32$	$4.72^{c} \pm 1.16$	$5.91^{b} \pm 1.12$	$6.92^{ab} \pm 0.96$	$7.72^{a} \pm 0.71$
	6	$4.37^{d} \pm 1.82$	$5.03^{cd} \pm 1.62$	$6.07^{bc} \pm 1.55$	$7.26^{ab} \pm 1.28$	$8.26^{a} \pm 1.01$
	12	$4.29^{d} \pm 1.57$	$4.84^{cd} \pm 1.20$	$5.79^{bc} \pm 1.19$	$6.76^{ab} \pm 1.06$	$7.78^{a} \pm 1.26$
Color uniformity	1	$8.34^{a} \pm 1.09$	$7.57^{ab} \pm 1.04$	$6.84^{b} \pm 1.25$	$6.16^{bc} \pm 1.49$	$5.19^{c} \pm 1.78$
	6	7.66 ± 1.66	7.36 ± 1.36	6.84 ± 1.54	6.53 ± 1.85	6.13 ± 1.99
	12	$7.16^{a} \pm 2.11$	$6.81^{ab} \pm 1.78$	$5.74^{abc} \pm 1.43$	$5.25^{bc} \pm 1.36$	$4.93^{c} \pm 1.28$
Hardness	1	3.78 ± 1.06	4.33 ± 0.89	4.51 ± 0.99	4.76 ± 1.15	4.61 ± 1.38
	6	4.44 ± 1.10	4.48 ± 1.06	4.81 ± 1.33	4.76 ± 1.64	4.95 ± 1.89
	12	4.76 ± 0.97	4.38 ± 1.20	4.88 ± 1.19	5.08 ± 1.80	5.10 ± 1.87
Juiciness	1	$6.13^{a} \pm 2.09$	$6.03^{a} \pm 1.58$	$5.69^{ab} \pm 1.13$	$5.10^{ab} \pm 0.92$	$4.39^{b} \pm 1.32$
	6	$6.09^{a} \pm 1.54$	$6.03^{a} \pm 1.36$	$5.69^{a} \pm 1.33$	$5.10^{ab} \pm 1.03$	$4.15^{b} \pm 1.26$
	12	$6.01^{a} \pm 1.26$	$6.10^{a} \pm 1.14$	$5.47^{ab} \pm 1.13$	$5.01^{ab} \pm 1.17$	$4.32^{b} \pm 1.40$
Graininess	1	$2.67^{d} \pm 1.62$	$3.25^{cd} \pm 1.53$	$4.56^{cb} \pm 1.47$	$5.51^{ab} \pm 1.60$	$6.76^{a} \pm 2.05$
	6	$2.14^{d} \pm 0.82$	$2.56^{cd} \pm 0.87$	$4.14^{bc} \pm 1.36$	$5.21^{ab} \pm 2.00$	$6.54^{a} \pm 2.17$
	12	$2.55^{c} \pm 1.71$	$2.48^{c} \pm 1.27$	$3.78^{bc} \pm 1.84$	$5.01^{ab} \pm 1.68$	$6.23^{a} \pm 2.09$
Binding	1	5.85 ± 1.90	6.62 ± 1.50	6.66 ± 1.63	6.77 ± 1.72	6.21 ± 1.76
	6	6.22 ± 1.80	6.36 ± 1.47	6.57 ± 1.68	6.29 ± 1.72	6.33 ± 1.92
	12	6.44 ± 1.54	6.08 ± 0.94	6.35 ± 1.22	5.98 ± 1.22	5.45 ± 1.83
Fatty-oil odour	1	2.81 ± 1.41	2.75 ± 1.55	2.84 ± 1.60	2.99 ± 1.96	3.08 ± 2.20
	6	4.22 ± 2.38	4.13 ± 2.41	3.81 ± 2.48	3.57 ± 2.46	3.43 ± 2.86
	12	4.32 ± 2.53	4.00 ± 2.33	3.53 ± 2.15	3.07 ± 2.16	2.80 ± 2.01
Meaty odour	1	$6.58^{a} \pm 2.24$	$6.13^{ab} \pm 2.22$	$5.56^{ab} \pm 2.20$	$5.05^{ab} \pm 2.01$	$4.15^{b} \pm 1.96$
	6	6.09 ± 1.75	5.76 ± 1.84	5.53 ± 2.10	4.83 ± 1.98	4.05 ± 1.99
	12	6.21 ± 2.00	5.88 ± 1.81	5.18 ± 1.81	4.41 ± 2.12	4.12 ± 2.03
Spicy odour	1	3.02 ± 1.60	3.26 ± 1.73	3.89 ± 2.01	4.02 ± 2.17	4.24 ± 2.25
	6	3.11 ± 2.02	3.12 ± 2.04	3.56 ± 1.86	3.60 ± 1.97	3.64 ± 2.59
	12	2.56 ± 0.98	2.73 ± 0.82	3.27 ± 1.13	3.07 ± 1.85	2.97 ± 2.16
Meaty taste	1	$7.86^{a} \pm 1.25$	$7.31^{a} \pm 1.43$	$6.81^{ab} \pm 0.98$	$5.64^{bc} \pm 1.69$	$5.09^{c} \pm 2.18$
	6	$7.16^{a} \pm 1.36$	$6{,}81^{a} \pm 1.32$	$6.06^{ab} \pm 1.29$	$5.24^{bc} \pm 1.01$	$4.01^{c} \pm 1.10$
	12	$7.00^{a} \pm 1.33$	$6.91^{a} \pm 1.15$	$6.33^{ab} \pm 1.44$	$5.37^{ab} \pm 1.78$	$4.64^{b} \pm 1.97$
Salty taste	1	4.69 ± 1.54	4.69 ± 1.49	4.77 ± 1.26	4.70 ± 1.23	4.44 ± 1.32
	6	4.80 ± 1.67	4.76 ± 1.55	4.59 ± 1.58	4.56 ± 1.68	4.52 ± 1.95
	12	4.80 ± 1.90	4.71 ± 1.79	4.59 ± 1.58	4.32 ± 1.50	4.16 ± 1.52
Fatty-oil taste	1	3.76 ± 2.23	3.76 ± 2.22	3.76 ± 2.21	3.71 ± 2.30	3.72 ± 2.42
	6	3.75 ± 2.05	3.32 ± 1.69	3.39 ± 2.08	3.34 ± 2.29	3.36 ± 2.67
	12	3.69 ± 1.91	3.66 ± 1.84	3.26 ± 1.71	3.48 ± 1.99	3.58 ± 2.18
Bitter taste	1	1.31 ± 0.52	1.42 ± 0.54	1.96 ± 1.41	2.15 ± 1.71	2.46 ± 2.25
	6	1.46 ± 0.73	1.61 ± 0.96	1.92 ± 1.47	2.04 ± 1.56	2.43 ± 2.25
	12	1.28 ± 0.36	1.52 ± 0.85	1.68 ± 0.71	1.68 ± 0.69	2.07 ± 1.40
Spicy/ herbal taste	1	3.94 ± 2.09	4.11 ± 1.82	4.75 ± 1.66	4.97 ± 1.50	4.94 ± 2.23
	6	4.20 ± 2.03	4.39 ± 1.93	4.89 ± 1.62	5.06 ± 1.85	5.47 ± 2.23
	12	3.70 ± 1.93	3.96 ± 1.62	4.54 ± 2.04	4.13 ± 2.19	3.88 ± 2.11
Overall score	1	$7.17^{ab} \pm 1.74$	$8.11^{a} \pm 1.23$	$7.44^{ab} \pm 1.46$	$5.93^{bc} \pm 1.61$	$4.86^{c} \pm 2.23$
	6	$7.14^{ab} \pm 1.17$	$7.94^{a} \pm 1.35$	$7.49^{a} \pm 1.40$	$5.89^{b} \pm 0.79$	$3.88^{c} \pm 1.29$
	12	$6.55^{ab} \pm 1.48$	$7.48^{a} \pm 1.77$	$6.97^{ab} \pm 1.74$	$5.26^{bc} \pm 1.74$	$3.52^{c} \pm 1.42$

^{a,b,c} – Means with different letters in a row for particular parameters indicate a significant effect of hemp cake addition (one-way ANOVA and *post hoc* Tukey test) ($P < 0.05$);
ns = non-significant; * = P < 0.05, ** = P < 0.01.

Table S5
Influence of the storage time (T), hemp cake addition (H), and their interaction on the sensory analysis of meatballs.

Parameter	Time (days) T				Hemp cake addition H	Interaction T x H
	1	6	12	p	p	p
Colour intensity	5.86 ± 1.72	6.20 ± 2.04	5.89 ± 1.77	ns	***	ns
Colour uniformity	6.82 ± 1.72[a]	6.91 ± 1.74[a]	5.98 ± 1.80[b]	**	***	ns
Hardness	4.40 ± 1.12	4.69 ± 1.41	4.84 ± 1.43	ns	ns	ns
Juiciness	5.47 ± 1.57	5.41 ± 1.47	5.36 ± 1.37	ns	***	ns
Graininess	4.55 ± 2.20	4.12 ± 2.23	4.01 ± 2.22	ns	***	ns
Binding	6.42 ± 1.69	6.35 ± 1.68	6.06 ± 1.43	ns	ns	ns
Fatty-oil odour	2.89 ± 1.72[b]	3.83 ± 2.47[a]	3.54 ± 2.24[ab]	*	ns	ns
Meaty odour	5.49 ± 2.23	5.25 ± 2.02	5.16 ± 2.06	ns	***	ns
Spicy odour	3.69 ± 1.97	3.47 ± 2.08	2.92 ± 1.45	ns	ns	ns
Meaty taste	6.54 ± 1.84[a]	5.86 ± 1.65[b]	6.05 ± 1.77[ab]	*	ns	ns
Salty taste	4.66 ± 1.34	4.65 ± 1.65	4.52 ± 1.63	ns	ns	ns
Fatty-oil taste	3.74 ± 2.21	3.43 ± 2.12	3.53 ± 1.87	ns	ns	ns
Bitter taste	1.86 ± 1.48	1.89 ± 1.49	1.65 ± 0.88	ns	*	ns
Spicy/herbal taste	4.54 ± 1.88	4.80 ± 1.94	4.04 ± 1.94	ns	ns	ns
Overal score	6.70 ± 2.01[a]	6.47 ± 1.89[ab]	5.96 ± 2.14[b]	*	ns	ns

[a,b,c] – means with different letters for particular parameters in a row indicate a significant effect of time (two-way ANOVA and *post hoc* Tukey test) ($P < 0.05$);
* = $p \leq 0.05$, ** = $p \leq 0.01$, *** = $p \leq 0.001$; ns = non-significant.
